# Contemporaneous Portal-Arterial Reperfusion during Liver Transplantation: Preliminary Results

**DOI:** 10.1155/2011/251656

**Published:** 2011-03-31

**Authors:** G. L. Adani, A. Rossetto, V. Bresadola, D. Lorenzin, U. Baccarani, D. De Anna

**Affiliations:** Department of Surgery and Transplantation, University Hospital of Udine, 33100 Udine, Italy

## Abstract

We prospectively compared sequential portal-arterial revascularization (SPAr, group 1 no. 19) versus contemporaneous portal-hepatic artery revascularization (CPAr, group 2 no. 21) in 40 consecutive liver transplantation (LT). There were no differences in the demographics characteristics, MELD score, indication to LT, and donor's parameters between the two groups. CPAr had longer warm ischemia 66 ± 8 versus 37 ± 7 min (*P* < .001), while SPAr had longer arterial ischemia 103 ± 42 min (*P* = .0004). One-year patient's and graft survival were, respectively, 89% and 95% versus 94% and 100% (*P* = .29). At median followup of 13 ± 6 versus 14 ± 7 months biliary complications were anastomotic stenosis in 15% versus 19% (*P* = .78), and intrahepatic nonanastomotic biliary strictures in 26% versus none (*P* = .01), respectively, in SPAr and CPAr. CPAr reduces the incidence of intrahepatic biliary strictures by decreasing the duration of arterial ischemia.

## 1. Introduction

Sequential portal-arterial revascularization (SPAr) of the graft is the most common technique in liver transplantation (LT), based on the experience that portal blood flow is sufficient for adequate liver function [1, 2]. The main disadvantage of SPAr might be an increased risk of warm ischemic damage to the bile ducts, which depend solely on arterial blood supply [3]. Some authors have advocated the use of contemporaneous portal-hepatic artery revascularization (CPAr) reporting no different parameters of early graft function, with less intrahepatic nonanastomotic biliary damages [1, 4]. In our study we prospectively compared SPAr versus CPAr in terms of graft function, reperfusion syndrome, vascular complications, one-year patient's and graft survival, and biliary complications analyzed as pureanastomotic versus intrahepatic nonanastomotic biliary strictures.

## 2. Patients and Methods

From June 2008 to December 2009, 40 consecutive LT from heart-beating donors were randomized 1 : 1 to SPAr (group 1, no. 19) or CPAr (group 2, no. 21). No differences between the groups for indications to OLT have been evidenced during statistical analysis. Cases requiring an aortohepatic bypass for arterial revascularization, and reOLT were excluded from the analysis. The LT was always carried out by piggy-back technique in all cases. The bile duct was reconstructed in all cases by termino-terminal duct to duct anastomosis; the T-tube was used, respectively, in 32% versus 29% of cases (*P* = .83). All grafts were whole liver except one extended right liver split including segment one in group 1. Portal vein and arterial anastomosis between the graft and the recipient have been always performed with terminal-terminal interrupted suture. In the SPAr group the graft was revascularized through the portal vein, and the hepatic artery was subsequently performed; the time interval between portal and arterial revascularization was recorded and defined as arterial ischemic time. In the CPAr the graft was revascularized simultaneously through the portal vein, and hepatic artery. Cold and warm ischemia time were determined, respectively, as the period between cross-clamp and graft inside the abdomen and between the graft in the abdomen and reperfusion. Reperfusion syndrome was defined as a drop in mean arterial pressure greater than 30% within 1 minute from reperfusion of the graft and lasting more than 5 minutes. Primary nonfunction (PNF) was defined as the need for retransplantation or death due to graft failure within 7 days in absence of technical problems. Delayed graft function (DGF) was defined as AST or ALT peak greater than 2000 U/ml within 72 hours from LT. Biliary complications were analyzed when clinically evident or through magnetic resonance cholangiopancreatography (MRCP) every 6 months after transplantation in asymptomatic patients. 

The type of reperfusion during evaluation of MRCP of the biliary tree was unknown to the radiologist. Moreover radiologists and gastroenterologists were also blinded regarding any differences in graft reperfusion, during treatment of symptomatic stenosis. 

 Statistical analysis was performed by the student's *t*-test or chi-square test as appropriated. A *P* value less than .05 was considered significant.

## 3. Results

**Figure 1:**
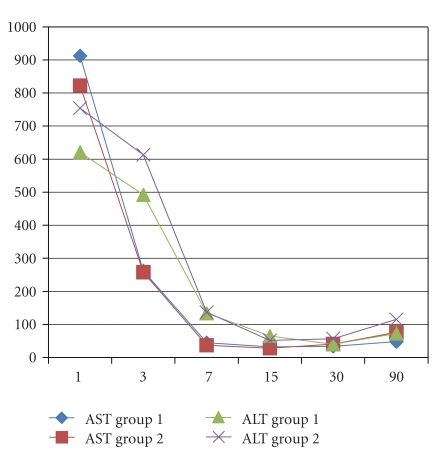


**Figure 2:**
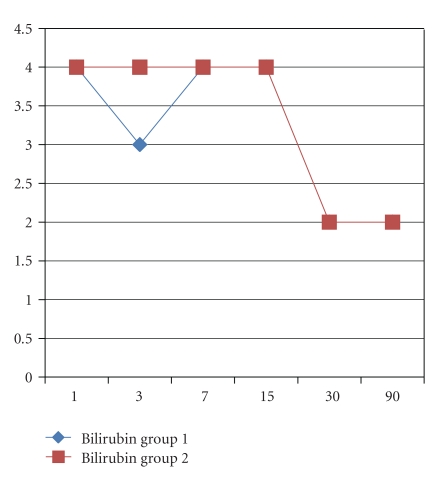


**Figure 3:**
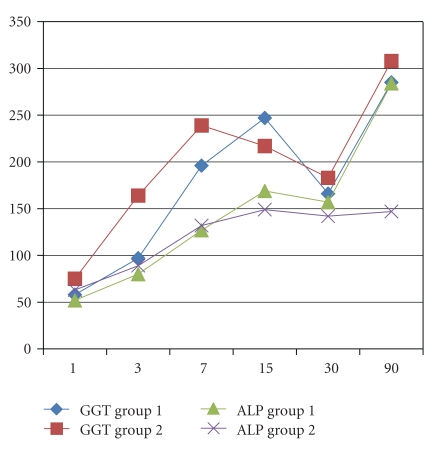


**Figure 4:**
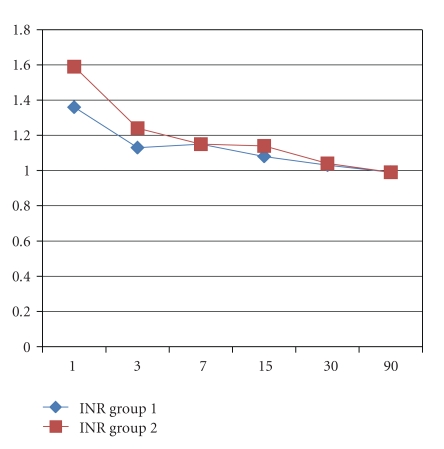


Recipient's age and male gender were, respectively, 52 ± 9 versus 56 ± 9 (*P* = .15) years old and 74% versus 75% (*P* = .93) in group 1 and 2. No differences in the indication for LT were encountered with HCC as the main indication in 32% versus 42%, HCV in 26% versus 24%, and alcohol in 16% versus 19% (*P* = ns). The MELD score was 17 ± 7 versus 14 ± 5 (0.12). Donor's age was, respectively, 52 ± 18 versus 51 ± 15 years old in groups 1 and 2 (*P* = .85). Twenty-one percent of the grafts in groups 1 versus 19% in group 2 had macrovacuolar steatosis greater than 15% (*P* = .92). Duration of LT was not different between groups 1 and 2 (392 ± 115 versus 373 ± 60 min, *P* = .52) as well as cold ischemia time (478 ± 147 versus 441 ± 88 min, *P* = .32), while warm ischemia time was statistically significant longer in the CPAr group (37 ± 7 versus 66 ± 8 min, *P* < .001). Arterial ischemic time in the SPAr group was statistically significant longer than warm ischemia time in the CPAr group (103 ± 42 versus 66 ± 8 min, *P* = .0004). Units of blood and amount of plasma transfusion were, respectively, 6 ± 4 versus 7 ± 4 (*P* = .60) and 1,300 ± 1,175 ml versus 2,382 ± 1,853 ml (*P* = .045) in groups 1 and 2. There were no PNF in both groups; DGF was diagnosed in 10% versus 9% in groups 1 and 2 (*P* = .91). ICU and total hospital stay were, respectively, 7 ± 4 versus 5 ± 2 and 17 ± 5 versus 18 ± 6 days, respectively, in groups 1 and 2 (*P* = .12 and *P* = .68) (Table 1). AST and ALT (Figure 1), total bilirubin, INR, GGT and ALP (Figures 2, 3, and 4), at days +1, +3, +7, +15, +30 and +90, did not evidenced any statistically significant difference between SPAr and CPAr. Vascular complications were absent except for one case of hepatic artery thrombosis in group 1 leading to retransplantation. One-year graft and patient's survival were, respectively, 95% versus 100% and 89% versus 94% in groups 1 and 2 (*P* = .29 and *P* = .53). At a median follow-up of 13 ± 6 versus 14 ± 7 months (*P* = .71) biliary complications diagnosed were anastomotic stenosis in 15% versus 19% (*P* = .78), and intrahepatic nonanastomotic biliary strictures in 26% versus none (*P* = .01), respectively, in SPAr and CPAr.

In all cases biliary stenosis occurred within 1 year after OLT, and these were successfully treated through ERCP when anastomotic (7 cases, 3 out of groups 1 and 4 out of group 2) and through PTC (5 cases out of group 1) when intrahepatic nonanastomotic strictures have been diagnosed.

## 4. Discussion

The most commonly used procedure for revascularization of the liver graft is initial reperfusion via the portal vein and subsequent reconstruction of the hepatic artery [5–7]. The disadvantage is that portal venous blood alone in a progressively rewarming graft may induce ischemic damage to the biliary tract, which depends solely on the hepatic artery blood supply. CPAr in LT has theoretically some potential advantages on SPAr: (a) the liver receives a larger blood supply during the critical phase of reperfusion injury; (b) arterial warm ischemia of the graft biliary tree is reduced; and (c) arterial anastomosis is performed in a surgical field without hemorrhage. The disadvantage of CPAr is longer warm ischemia time and the anhepatic phase, which can be detrimental to postoperative graft function, survival, and morbidity [8, 9]. In our study, recovery of graft function, and patient and graft survival rates was similar in both groups. Similar results were reported in two previous clinical studies that compared CPAr and SPAr with regard to different parameters of early graft function [6–8]. A delay in reestablishing arterial inflow in an exclusively portal perfused liver potentially prolongs the warm ischemia time to the bile ducts contributing to the development of biliary strictures. Non-anastomotic biliary stenosis has been associated with hepatic artery thrombosis or stenosis and with prolonged cold and warm ischemia time [10, 11]. Even if a longer warm ischemia time can be a possible risk factor for biliary strictures [8], it has not been evidenced in our cases. In our study the arterial warm ischemia time in the control group (SPAr) was statistically significant longer than warm ischemia time in the CPAr group confirming a possible greater amount of selective arterial ischemia to the bile duct in the group with sequential portal and arterial revascularization. This resulted in a higher incidence of nonanastomotic intrahepatic biliary strictures in the SPAr group (26% versus none) thereby suggesting a possible protective role of CPAr on the integrity of the intrahepatic biliary tree potentially due to less selective arterial ischemia.

## Figures and Tables

**Table 1:** 

	Group 1	Group 2	*P*
Duration of surgery	392 ± 115	373 ± 60	.52
Cold ischemia time	478 ± 147	441 ± 88	.32
Warm ischemia time	37 ± 7	66 ± 8	<.0001
Units of blood transfused	6 ± 4	7 ± 4	.60
Plasma transfused (ml)	1300 ± 1175	2382 ± 1853	.045
Postreperfusion syndrome	2	5	.26
ICU stay (day)	7 ± 4	5 ± 2	.12
Total hospital stay (day)	17 ± 5	18 ± 6	.68
Biopsy-proven acute rejection	2	1	.48
Portal complications	0	0	n.a
Arterial complications (thrombosis)	1	0	.76
Biliary complications	9	4	.05
PNF	0	0	n.a.
DGF	4	2	.91
One-year graft survival	18	21	.29
One-year patient survival	17	20	.53
Followup (months)	13 ± 6	14 ± 7	.71
